# Neuropharmacological Effects of the Dichloromethane Extract from the Stems of *Argemone ochroleuca* Sweet (Papaveraceae) and Its Active Compound Dihydrosanguinarine

**DOI:** 10.3390/ph16081175

**Published:** 2023-08-18

**Authors:** Eunice Yáñez-Barrientos, Juan Carlos Barragan-Galvez, Sergio Hidalgo-Figueroa, Alfonso Reyes-Luna, Maria L. Gonzalez-Rivera, David Cruz Cruz, Mario Alberto Isiordia-Espinoza, Martha Alicia Deveze-Álvarez, Clarisa Villegas Gómez, Angel Josabad Alonso-Castro

**Affiliations:** 1 Departamento de Química, División de Ciencias Naturales y Exactas, Universidad de Guanajuato, Noria Alta, Colonia Noria Alta Guanajuato, Guanajuato 36050, Mexico; eybarrientos@ugto.mx (E.Y.-B.); a.reyes.luna@ugto.mx (A.R.-L.); david.cruz@ugto.mx (D.C.C.); 2 Departamento de Farmacia, División de Ciencias Naturales y Exactas, Universidad de Guanajuato, Noria Alta, Colonia Noria Alta Guanajuato, Guanajuato 36050, Mexico; jcbarragang@gmail.com (J.C.B.-G.); leonor.glez.rivera@outlook.com (M.L.G.-R.); devezem@ugto.mx (M.A.D.-Á.); 3 CONAHCyT-División de Biología Molecular, Instituto Potosino de Investigación Científica y Tecnológica A.C., San Luis Potosí 78216, Mexico; sergio.hidalgo@ipicyt.edu.mx; 4 Instituto de Investigación en Ciencias Médicas, Departamento de Clínicas, División de Ciencias Biomédicas, Centro Universitario de los Altos, Universidad de Guadalajara, Tepatitlán de Morelos 47620, Jalisco, Mexico; mario.isiordia162@yahoo.com

**Keywords:** *Argemone ochroleuca* Sweet, anxiolytic, antidepressant, anticonvulsant

## Abstract

*Argemone ochroleuca* Sweet (Papaveraceae) is used in folk medicine as a sedative and hypnotic agent. This study aimed to evaluate the anxiolytic-like, sedative, antidepressant-like, and anticonvulsant activities of a dichloromethane extract of *A. ochroleuca* stems (AOE), chemically standardized using gas chromatography–mass spectrometry (GC–MS), and its active compound dihydrosanguinarine (DHS). The anxiolytic-like, sedative, antidepressant-like, and anticonvulsant activities of the AOE (0.1–50 mg/kg p.o.) and DHS (0.1–10 mg/kg p.o.) were evaluated using murine models. A possible mechanism for the neurological actions induced by the AOE or DHS was assessed using inhibitors of neurotransmission pathways and molecular docking. Effective dose 50 (ED_50_) values were calculated by a linear regression analysis. The AOE showed anxiolytic-like activity in the cylinder exploratory test (ED_50_ = 33 mg/kg), and antidepressant-like effects in the forced swimming test (ED_50_ = 3 mg/kg) and the tail suspension test (ED_50_ = 23 mg/kg), whereas DHS showed anxiolytic-like activity (ED_50_ = 2 mg/kg) in the hole board test. The AOE (1–50 mg/kg) showed no locomotive affectations or sedation in mice. A docking study revealed the affinity of DHS for α2-adrenoreceptors and GABA_A_ receptors. The anxiolytic-like and anticonvulsant effects of the AOE are due to GABAergic participation, whereas the antidepressant-like effects of the AOE are due to the noradrenergic system. The noradrenergic and GABAergic systems are involved in the anxiolytic-like actions of DHS.

## 1. Introduction

*A. ochroleuca* Sweet (Papaveraceae), a native plant from México and widely distributed in America and Australia, is a perennial herb, flowers from February to May, and can grow up to 1.2 m in height, mainly near highways or brownfields. *Argemone ochroleuca*, commonly known as “chicalote” or “cardo santo”, is used in Mexican folk medicine as an antitussive, anticonvulsant, antidiarrheal, antispasmodic, hypnotic, narcotic, sedative, and analgesic. Additionally, it is useful for treating eye infections, eye spots, and dermatoses [1,2,3,4,5,6,7].

**Figure 1:**
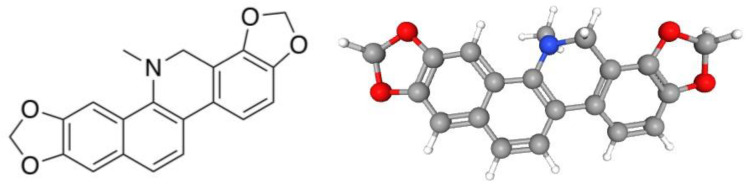
Chemical structures (2D and 3D) of dihydrosanguinarine.

The raw latex of *A. ochroleuca* showed antibacterial effects against enteropathogenic bacteria and antifungal activity against *Drechslera halodes* and four *Candida* species [8,9]. The methanolic extract of the aerial parts of *A. ochroleuca* showed antimicrobial activity against *Staphylococcus aureus* (minimum inhibitory concentration, MIC = 125 mg/mL) and *Cryptococcus neoformans* (MIC = 500 mg/mL) [10]. Previous studies reported the antibacterial activity of the ethanolic extracts of the aerial parts of *A. ochroleuca* against *S. aureus* and *Bacillus subtilis* [11,12], the antiparasitic effects of the chloroform fraction from the aerial parts of *A. ochroleuca* against *Plasmodium falciparum* (inhibitory concentration 50, IC_50_ = 0.32 mg/mL) and *Trypanosoma cruzi* (IC_50_ = 0.30 mg/mL), and the low toxicity against MRC-5 cells (IC_50_ = 11.6 mg/mL) [13]. In silico studies revealed that *A. ochroleuca* is a source of compounds with antidiabetic activity [14]. On the other hand, the dichloromethane extract of the aerial parts of *A. ochroleuca* showed relaxant activity in the smooth muscle of guinea pig (effective concentration 50, EC_50_ = 78.03 ± 2.15 mg/mL) [5]. Alkaloids, such as tetrahydroberberine, allocryptopine, and protopine, are abundant in members of the Papaveraceae family. These compounds have been identified in *A. ochroleuca* and other plant species [5,15,16,17], and exert neuroprotective effects [18]. Dihydrosanguinarine (DHS) (Figure 1), a benzophenanthridine alkaloid isolated from different plant species of the Papaveraceae family, showed antimicrobial activity against *S. aureus* and *Streptococcus faecalis* (MIC = 9.3 µg/mL) [19], cytotoxic and antiproliferative effects against cancer cell lines [20,21], and in vitro antidiabetic actions [22]. DHS (100–500 mg/kg p.o.) showed no significant alterations in body or organ weights in rats administered for 90 days [20]. Furthermore, no alterations in biochemical parameters (e.g., AST, ALT, ALP, etc.) or hematological parameters (e.g., hematocrit, hemoglobin, etc.) were shown. The pharmacokinetic parameters of 91 ng/kg DHS were t_max_ = 1 h, c_max_ = 28.08 ng/mL, and AUC = 51.86 mg/mL h [20]. DHS exerted antinociceptive activity (ED_50_ = 85 mg/kg) in the formalin test in mice without inducing gastric damage and inducing low toxicity (LD_50_ > 2000 mg/kg i.p.). The antinociceptive activity of DHS was associated with the participation of GABAergic neurotransmission without showing sedative or neurotoxic effects [23].

Mental health conditions such as anxiety and depression affect millions of people worldwide [24]. The treatment for these conditions includes the use of psychotropic medications. However, the adverse reactions caused by these drugs decrease treatment adherence. In addition, many patients might not respond to the pharmacological treatment [24]. Medicinal plants such as *Cannabis sativa*, *Hypericum perforatum*, *Valeriana officinalis*, and others have shown clinical efficacy, and their active components have been identified and tested in clinical trials. Therefore, medicinal plants are a good source of active compounds for the pharmacological therapy of mental health conditions. Alkaloids are one of the most studied groups of secondary metabolites that have shown anxiolytic-like, antidepressant-like, sedative, and anticonvulsant actions in preclinical models [24]. However, DHS lacks studies of its neuropharmacological effects.

Due to its uses in Mexican folk medicine as a relaxant, sedative, and hypnotic, as well as the presence of alkaloids such as DHS, tetrahydroberberine, allocryptopine, and protopine [1,2,3,4,5,6,7], it is probable that *A. ochroleuca* might exert neuropharmacological effects. This study aimed to evaluate the anxiolytic-like, sedative, antidepressant, and anticonvulsant activities of the dichloromethane extract of the stems of *A. ochroleuca* (AOE) and its active compound DHS.

## 2. Results

### 2.1. Chemical Characterization of AOE 

**Figure 2:**
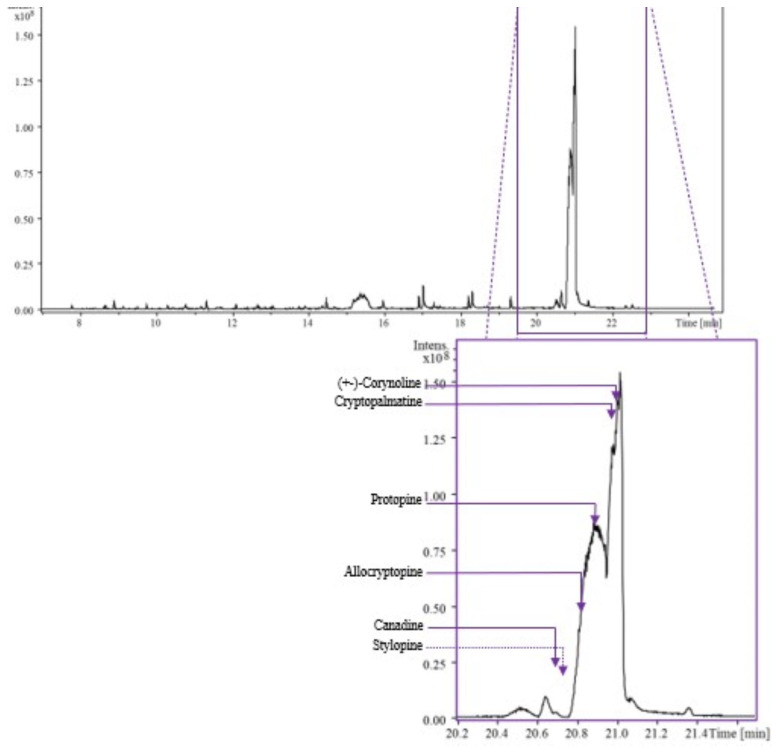
The chromatogram obtained during GC–MS analysis of AOE indicated the peaks registered at 330 ± 16 nm, under analytical conditions.

A total of 8.3 g of extract was obtained. The deconvoluted spectra and retention indexes were compared with those reported in libraries in order to identify each compound. Forty-four compounds were identified in the AOE, consisting of 17 aliphatic compounds, 8 alkaloids, 1 aromatic compound, 7 carboxylic acids and related compounds, 6 phenolics, 1 benzoquinone, and 4 terpenes (Table 1 and Figure 2).

For the identification of the main components in the AOE, signals were selected with a signal-to-noise ratio equal to or greater than 5. Peak identification was carried out based on the retention index and fragmentation patterns using NIST/EPA/NIH (NIST 2020) and Wiley 12th edition Mass Spectral Libraries. Abundance is not presented in Table 2 because the signal intensity in a GC–MS chromatogram depends on the detector’s response, which can vary for different compounds. In electro-ionization, the analyte molecules lead to fragmentation and the generation of ions. The ionization efficiency can vary significantly for different compounds, and without a standard, it is challenging to establish a consistent relationship between the intensity of the fragmented ions and the concentration of the compound of interest.

### 2.2. Acute Toxicity and Anxiolytic-like Activity

The acute toxicity of the AOE resulted in a lethal dose 50 (LD_50_) higher than 2000 mg/kg p.o., and the mice showed no visible signs of toxicity (i.e., immobility, piloerection, anorexia, unusual respiratory pattern, etc.) for 14 days after a single administration of the plant extract.

**Figure 3:**
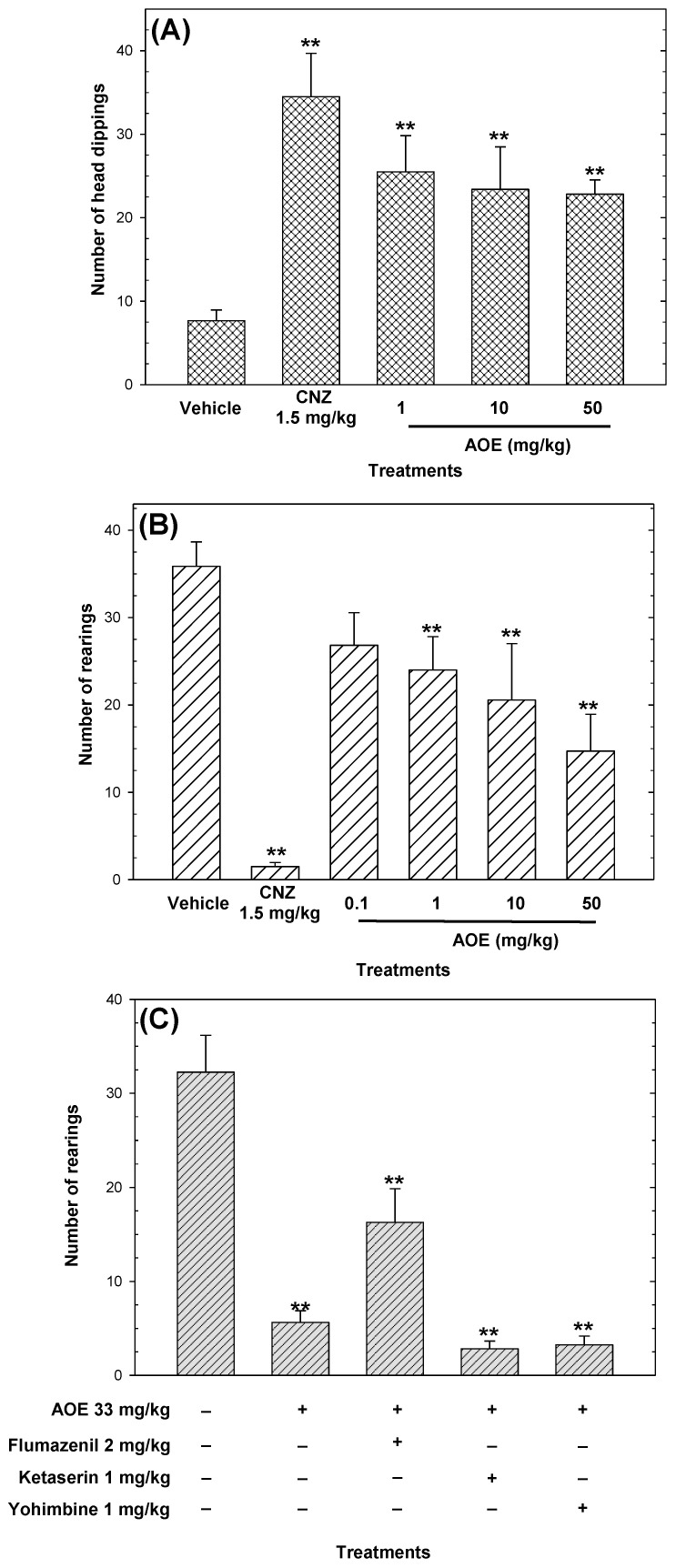
Anxiolytic-like effects of AOE measured by the number of head dippings (**A**) and the number of rearings (**B**) during 5 min of exposure. A possible mechanism of the anxiolytic-like effects of AOE was evaluated in the cylinder exploratory test (**C**). Clonazepam (CNZ) was the reference drug. Data are presented as mean ± standard error of the mean (n = 7 per group), ** *p* < 0.05 vs. the vehicle group, and ** *p* < 0.05 vs. the AOE 33 mg/kg p.o. group.

**Figure 4:**
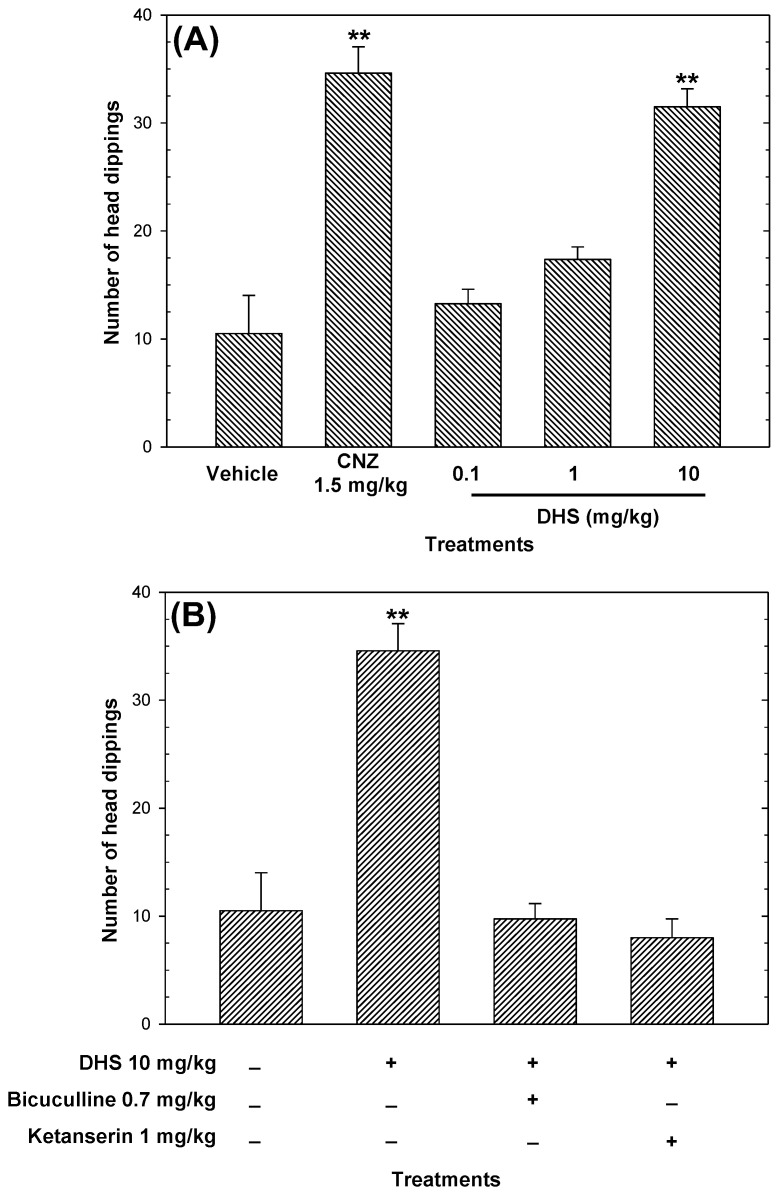
Anxiolytic-like effects of DHS (0.1–10 mg/kg p.o.) measured by the number of head dippings (**A**) during 5 min of exposure. A possible mechanism of the anxiolytic-like effects of DHS was evaluated in the hole board test (**B**). Clonazepam (CNZ) was the reference drug. Data are presented as mean ± standard error of the mean (n = 7 per group) and ** *p* < 0.05 vs. the vehicle group.

The AOE (Figure 3A) and DHS (Figure 4A) increased (*p* < 0.05) in an independent-dose manner the number of head dippings, compared to the vehicle. However, the AOE showed activity not equivalent to the positive control (Figure 3A), and the anxiolytic-like effects shown by DHS (ED_50_ *=* 2 mg/kg) were comparable to 1.5 mg/kg CNZ (Figure 4A). The maximum anxiolytic-like activity of the AOE was achieved at 50 mg/kg, whereas 1.5 mg/kg clonazepam (CNZ) also increased (*p* < 0.05) the occurrence of head dipping (Figure 3A).

In the cylinder exploratory test, the AOE decreased the number of rearings in a dose-dependent manner (ED_50_ *=* 33 mg/kg, calculated by linear regression analysis) with lower anxiolytic-like activity compared to 1.5 mg/kg CNZ. The pre-treatment with 2 mg/kg flumazenil attenuated (*p* < 0.05) the anxiolytic-like activity of the AOE in the cylinder exploratory test (Figure 3C). The pre-treatment with bicuculline and ketanserin abolished (*p* < 0.05) the anxiolytic-like effect of DHS in the head dipping test (Figure 4B).

### 2.3. Antidepressant-like Activity

**Figure 5:**
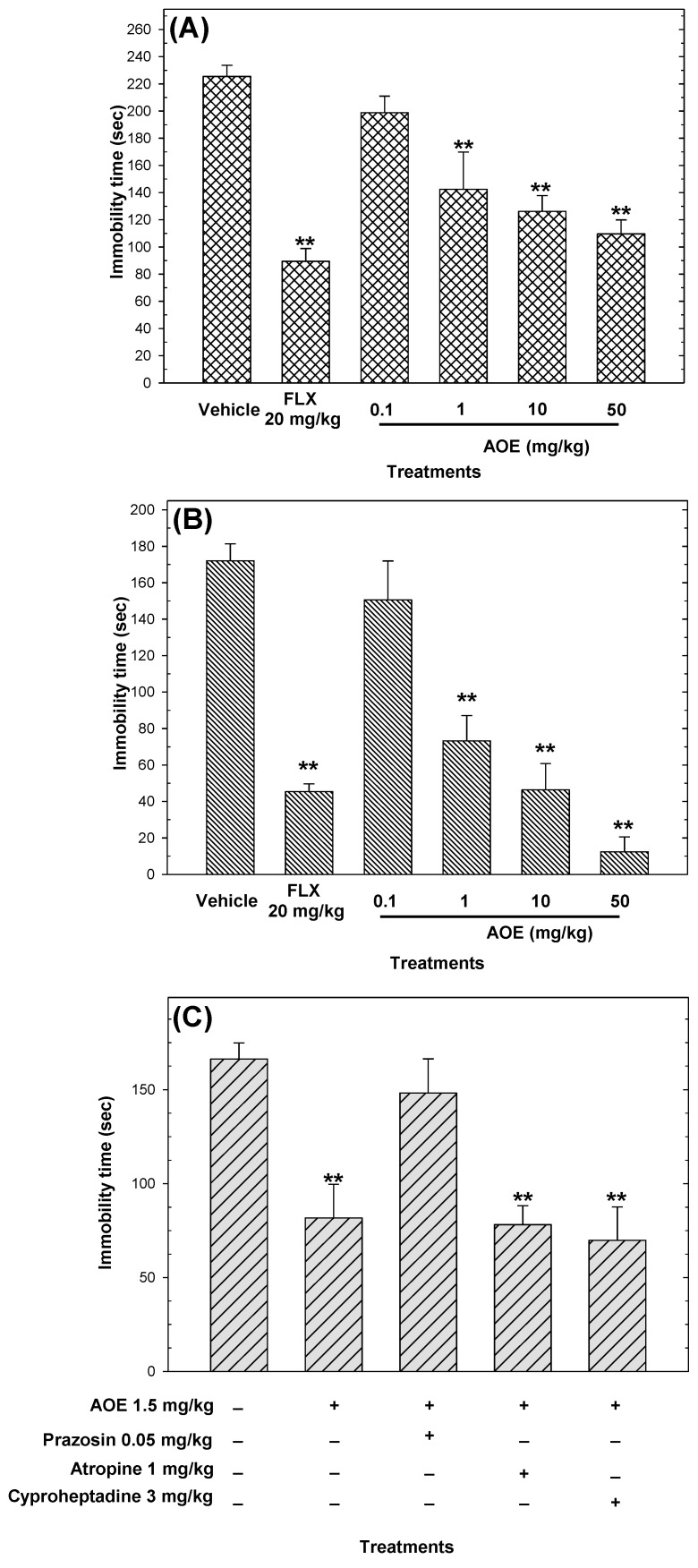
Antidepressant-like actions of AOE (0.1–50 mg/kg p.o.) in the tail suspension test (**A**) and the forced swimming test (**B**). The possible mechanism of action of AOE was evaluated in the forced swimming test (**C**). Fluoxetine (FLX) was the reference drug. Data are presented as mean ± standard error of the mean (n = 7 per group), ** *p* < 0.05 vs. the vehicle group.

**Figure 6:**
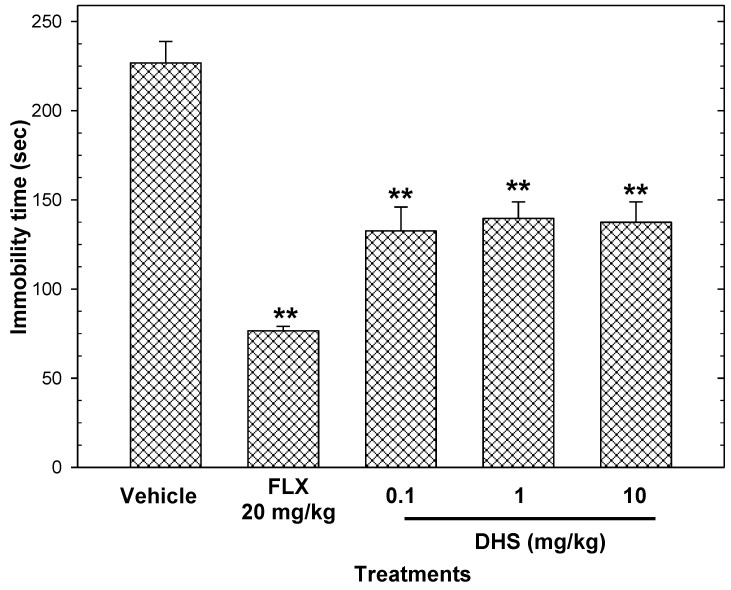
Antidepressant-like actions of DHS (0.1–10 mg/kg p.o.) in the tail suspension test. Fluoxetine (FLX) was the reference drug. Data are presented as mean ± standard error of the mean (n = 7 per group) and ** *p* < 0.05 vs. the vehicle group.

The AOE showed antidepressant-like activity in a dose-dependent manner in the forced swimming test and the tail suspension test (Figure 5). The antidepressant-like activity of the AOE was higher (*p* < 0.05, calculated by t-student test) in the forced swimming test (ED_50_ *=* 3 mg/kg) in comparison to the tail suspension test (ED_50_ *=* 23 mg/kg). DHS showed non-dependent antidepressant activity in the tail suspension test, and the reduction in immobility (described in Section 4.9) ranged from 38.42% to 41.51% (Figure 6).

The antidepressant activity of the AOE at 10 and 50 mg/kg (Figure 5) in the tail suspension test and the forced swimming test was comparable to that shown by 20 mg/kg FLX, whereas the antidepressant activity of DHS (0.1–10 mg/kg) showed no comparable activity to the reference drug in the tail suspension test (Figure 6). Co-administration with 0.05 mg/kg prazosin blocked the antidepressant-like actions of the AOE (Figure 5C).

### 2.4. Effects on Sedation and Motor Coordination

**Figure 7:**
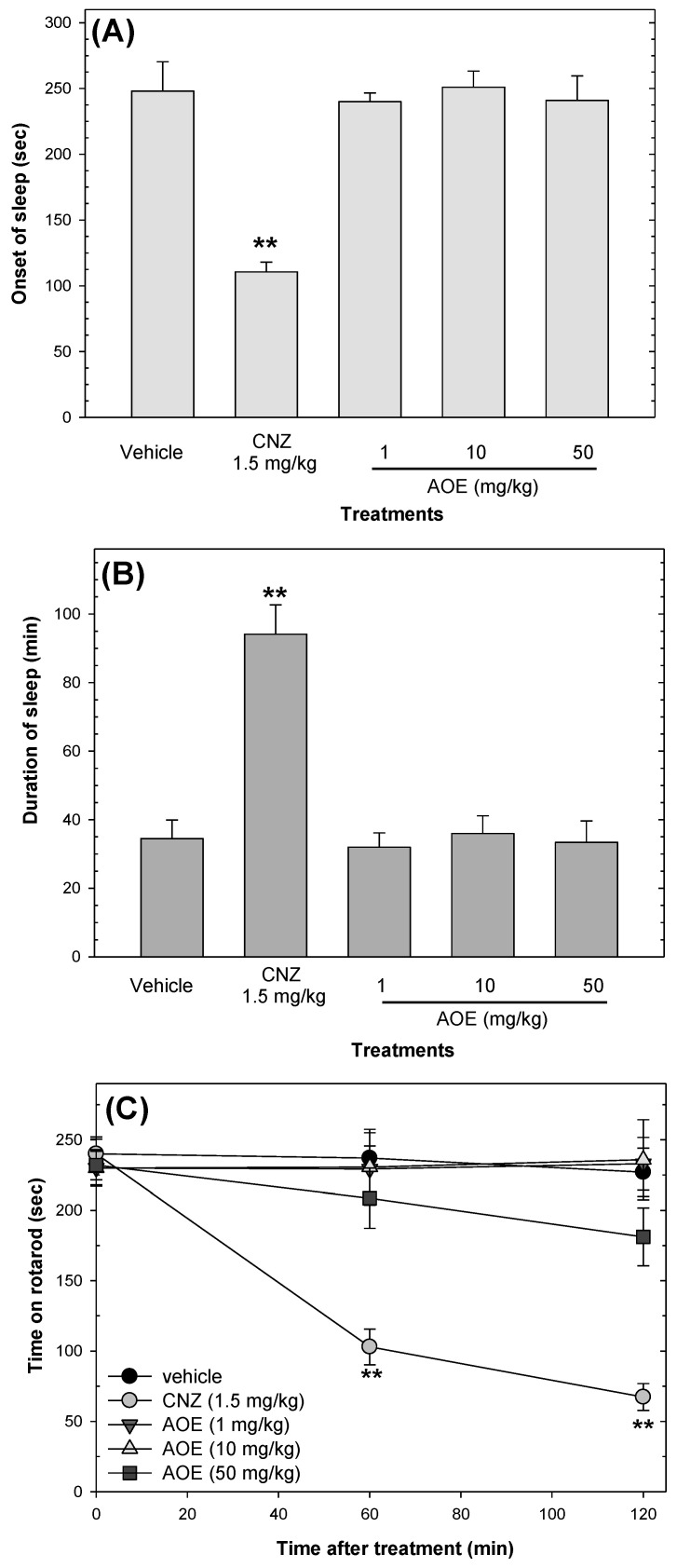
Effects of AOE on sedation and locomotor coordination. The pentobarbital-induced sleeping test measured the sedative effects of AOE on the onset of sleep (**A**) and the duration of sleep (**B**). The rotarod test assessed motor coordination (**C**). Clonazepam (CNZ) was the reference drug. Data are presented as mean ± standard error of the mean (n *=* 7 per group) and ** *p* < 0.05 vs. the vehicle group.

The AOE showed no sedative effects; only 1.5 mg/kg CNZ interfered (*p* < 0.05) with the locomotor activity of mice at 60 and 120 min post-treatment. CNZ decreased (*p* < 0.05) the onset of sleep and increased (*p* < 0.05) sleep duration compared to the vehicle group, whereas the AOE (1–50 mg/kg p.o.) did not affect the onset or duration of sleep (Figure 7).

### 2.5. Anticonvulsant Activity

In both models of convulsions, CNZ (1.5 mg/kg) prolonged (*p* < 0.05) the onset of convulsions and decreased (*p* < 0.05) the duration of seizures in comparison to the vehicle group (Table 2). Moreover, 1.5 mg/kg CNZ protected the mortality of mice treated with 2 mg/kg strychnine and 10 mg/kg 4-aminopyridine by 20% and 30%, respectively. In both models of convulsions, the AOE prolonged (*p* < 0.05) the onset of convulsions in a dose-dependent manner (Table 2).

In the strychnine-induced convulsion test, the AOE showed protection against mortality in a dose-dependent manner (Table 2). The pre-treatment with flumazenil reverted the delay in the onset of convulsions and the protection against seizures shown by 1 mg/kg AOE (Table 2).

In the 4AP-induced convulsion test, the different doses of DHS (0.1–10 mg/kg) increased (*p* < 0.05) the seizure duration compared to the vehicle, whereas 10 mg/kg DHS had a significant (*p* < 0.05) effect on delaying seizure onset.

### 2.6. Docking Studies

**Figure 8:**
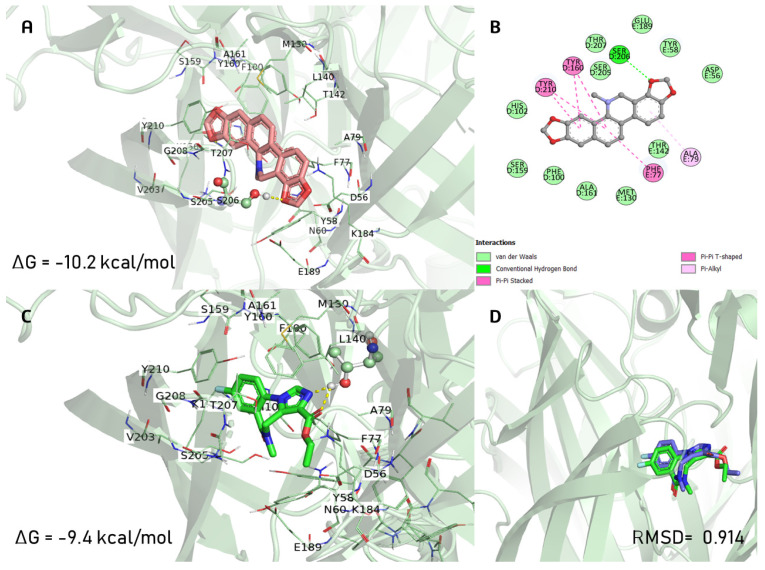
The docking complex of (**A**) dihydrosanguinarine (deep salmon color) with GABAA receptor and (**B**) 2D representations of molecular interactions. (**C**) Interactions of FYP (co-crystal ligand; green color) with GABAA receptor and (**D**) overlay of the co-crystallized pose (green) and the re-docked pose (blue) from validation (RMSD = 0.914 Å).

DHS exhibited the same orientation mode as the co-crystal ligand (FYP) on the GABA_A_ receptor. Interestingly, the binding involved more π-π and π-alkyl interactions with residues Phe77, Ala79, Tyr160, Tyr210, and only one hydrogen bond with Ser206 (Figure 8B). Instead, FYP preferred to form a dual hydrogen bond with Thr142 (Figure 8C). In comparison to the agonist FYP (ΔG *=* −9.4 kcal/mol), the binding energy for DHS was −10.2 kcal/mol, and DHS shared the ligand recognition site and shape of FYP for the GABA_A_ receptor.

**Figure 9:**
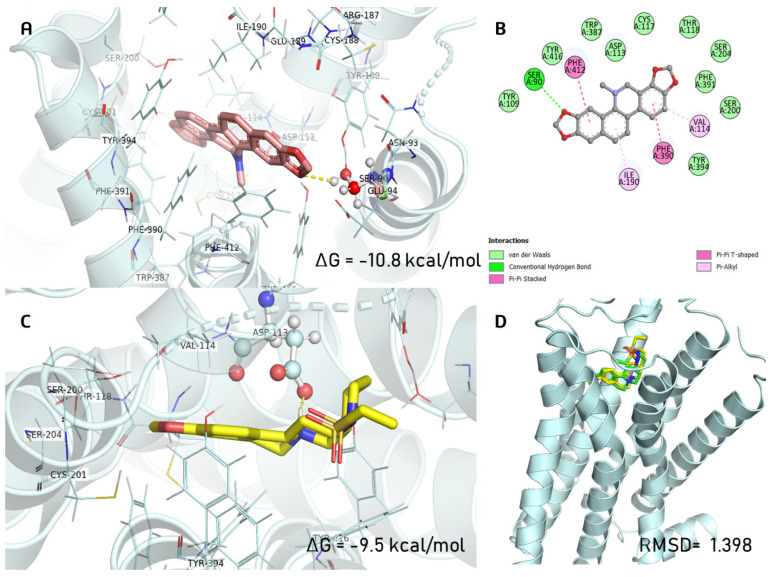
The docking complex of (**A**) dihydrosanguinarine (deep salmon color) with α_2A_-adrenergic receptor and (**B**) 2D representations of molecular interactions. (**C**) Interactions of E3F (co-crystal ligand; yellow color) with α_2A_-adrenergic receptor and (**D**) overlay of the co-crystallized pose (yellow) and the re-docked pose (green) from validation (RMSD = 1.398 Å).

For the α_2A_-adrenergic receptor, DHS showed a different orientation mode, including adopting an outward position and anchoring to Ser90 by a hydrogen bond and being strengthened by interactions with the Val114, Ile190, Phe390, and Phe412 residues (Figure 9A). E3F was anchored to Asp113 with a free binding energy of ΔG *=* −9.5 kcal/mol, and the binding energy for DHS was −10.8 kcal/mol.

### 2.7. ADMET Properties of DHS

DHS, FYP, and E3F showed a high probability of crossing the central nervous system barrier, being absorbed by the human intestine, and not being potential substrates or inhibitors for P-glycoprotein (P-gp). Finally, all cytochrome P450 isoforms could metabolize DHS (Table 3).

## 3. Discussion

This study evaluated the neurological actions of a dichloromethane extract from the stems of *A. ochroleuca* and explored the chemical composition of the plant extract by GC–MS. Furthermore, this study described the neuropharmacological (anxiolytic-like, antidepressant-like, sedative, and anticonvulsant) effects of the plant extract. The neuropharmacological actions of DHS, one active compound of *A. ochroleuca* with no previous neuropharmacological actions, were also assessed. The possible mechanism of the anxiolytic-like action of DHS was evaluated using inhibitors of neurotransmission and confirmed by an in silico study.

Previous reports studied the chemical characterization of *A. ochroleuca* and found compounds such as fatty acids, flavonoids, and alkaloids (dihydrosanguinarine, dihydrochelerythrine, and berberine) [5,15,16,17]. This study also found the presence of dihydrosanguinarine in the AOE using GC–MS. *A. ochroleuca* latex, contained in the stems, is employed in traditional medicine [8,9]. We selected working with *A. ochroleuca* stems to avoid a decrease in the plant population. We performed a chemical characterization using GC–MS of different plant parts of *A. ochroleuca*, including the stem, leaves, and branches, using ethanol and dichloromethane for extraction (results not shown). We only presented in the manuscript the plant extract for which we could identify more secondary metabolites of pharmacological interest, including alkaloids.

Clonazepam, an inhibitor of γ-aminobutyric acid (GABA), is used for the acute treatment of panic disorder, insomnia, and certain types of seizures [25]. It can be administered alone or in combination with other psychiatric drugs. CNZ was used as a positive control due to its anxiolytic, sedative, and anticonvulsant effects [25]. In acute administration, CNZ induces motor and cognitive impairment, sleep disorders, and the aggravation of mood. Prolonged use of CNZ produces physical dependence and tolerance [26]. Fluoxetine, a selective serotonin reuptake inhibitor, is used to treat major depressive disorder, obsessive–compulsive disorder, and panic disorder [26]. FLX was the positive control in the depression-induced assays. During the first months of treatment, fluoxetine might induce nausea, insomnia, nervousness, and somnolence, whereas the long-term administration of fluoxetine might induce cardiovascular complications and dyskinesia [26]. There is a great need to find new anxiolytic and antidepressant drugs with few adverse effects.

People use medicinal plants as a complementary therapy for treating anxiety and depression [24]. Nevertheless, many medicinal plants lack scientific evidence of their reputed use as anxiolytics and antidepressant agents.

A low frequency of head dipping in the hole board test and a low number of rearings in the cylinder exploratory test indicates anxiety-like behavior [27,28]. The AOE decreased the number of rearings in a dose-dependent manner in the cylinder exploratory test. The AOE, in a dose-independent action, and DHS, in a dose-dependent way, increased the number of head dippings in the hole board test. In addition, 10 mg/kg DHS showed similar anxiolytic-like activity compared to 1.5 mg/kg CNZ in the hole board test. These findings indicate that the AOE and DHS exerted anxiolytic-like effects.

The tail suspension test causes immobility by providing an inescapable place for mice and mimics depressive behavior [29]. The forced swimming test restricts space with no escape, which simulates a desperate situation, leading to immobility in rodents [30]. The AOE decreased immobility over time in mice in the forced swimming test and the tail suspension test; the effect of 50 mg/kg was similar to that shown by 20 mg/kg FLX. This activity suggests antidepressant-like actions. On the contrary, DHS showed antidepressant-like actions in a non-dose-dependent way, with lower activity compared to the reference drug.

Potentiation of the hypnotic effect of pentobarbital, a barbituric drug, is an indication of sedative activity [31]. The rotarod test can directly evaluate motor coordination and indirectly evaluates strength and sedation [32]. The AOE did not induce sedation or affect locomotor coordination in mice.

Strychnine is a glycine receptor antagonist [33], whereas 4-aminopyridine causes seizures by inhibiting voltage-dependent K^+^ channels [34]. The findings indicate that the AOE induced its anticonvulsant effects by the possible participation of the glycinergic and GABAergic systems.

The findings showed that the AOE exerted its anxiolytic and antidepressant actions through the possible participation of the GABAergic and noradrenergic systems, respectively. An impairment in the noradrenergic system is involved in developing depression and anxiety [35]. Imipramine, desipramine, protriptyline, and others are antidepressant drugs that act through the noradrenergic system. Lofepramine, a noradrenaline uptake inhibitor, is used to decrease the number of panic attacks [35,36]. The noradrenergic system is a therapeutic target for anxiety and depression [35,36].

Dihydrosanguinarine (DHS) was one of the compounds identified in the AOE. The participation of the GABAergic system in the antinociceptive actions of DHS was recently reported [23]. DHS (100 mg/kg i.p.) showed a moderate reduction in the ambulatory activity of mice, but no sedative, hypnotic, or neurotoxic effects were observed by an electroencephalographic analysis in mice [23]. The present study corroborates that DHS uses the GABAergic system for its pharmacological actions. Our results also present evidence, using neurotransmission inhibitors and a docking study, of the participation of the noradrenergic system in the anxiolytic-like actions of DHS. Serine is important, but not essential, in the α_2A_-adrenergic agonism. Several agonists form a hydrogen bond with S5.46 or S5.42, but many α_2A_-adrenergic agonists such as dexmedetomidine and clonidine do not interact with these mentioned residues. Therefore, our findings show for the first time that DHS has an alternative activation mechanism for α_2A_-adrenergic receptors [37,38]. The anxiolytic-like actions of the AOE, with the participation of the GABAergic system, were corroborated with the results, indicating that DHS induces anxiolytic-like activity through noradrenergic and GABAergic systems.

The most abundant compounds in the AOE were aliphatics and alkaloids, which agrees with previous studies [1,6,15,16]. Based on the GC–MS analysis, some other compounds that can contribute to the neuropharmacological effects shown by the AOE are the alkaloids tetrahydroberberine, allocryptopine, and protopine, which have been identified in *A. ochroleuca* and other plant species [5,15,16,17], and exert neuroprotective effects [18]. Tetrahydroberberine at 50 mg/kg decreased locomotor activity in mice by 80% but showed no sedative effects [39]. Tetrahydroberberine, considered a dopaminergic system antagonist [40], exerts neuroprotective effects through the blockage of ATP-sensitive potassium channels [41].

The results obtained for blood–brain barrier permeability showed a high probability of DHS, FYP, and E3F crossing the central nervous system barrier, increasing the possible absorption and distribution in the central nervous system. DHS, FYP, and E3F could be absorbed by the human intestine, allowing it to reach the site of action. DHS, FYP, and E3F did not seem to be inhibitors of P-glycoprotein (P-gp), a multidrug-resistant target that effluxes drugs and xenobiotics [42]. The inhibition of cytochrome P450 isoforms might cause drug–drug interactions in which co-administered drugs are unsuccessful in metabolizing the drug, thereby increasing its amount to toxic levels [42]. All of the cytochrome P450 isoforms could metabolize DHS, which decreased the possible toxicity from the co-administration with other drugs. DHS, FYP, and E3F did not exhibit inhibition over all cytochrome P450 isoforms, FYP and E3F did not show an affinity for any isoforms. All of the cytochrome P450 isoforms could metabolize DHS, which decreased the possible toxicity from the co-administration with other drugs. Finally, DHS showed low acute toxicity with an LD_50_ value greater than 500 mg/kg but less than 5000 mg/kg (Category IV), whereas E3F showed an LD_50_ value of 74 mg/kg (Class I).

Some of the limitations of this study include: (a) it was not possible to estimate the abundance of the compounds present in the AOE because the signal intensity in a GC–MS chromatogram depends on the detector’s response; (b) physiological experiments need to be carried out to elucidate the mechanisms of DHS.

## 4. Materials and Methods

### 4.1. Drugs

Clonazepam, fluoxetine, polyvinylpyrrolidone, yohimbine, flumazenil, ketanserin, atropine, cyproheptadine, prazosin, strychnine, and 4-aminopyridine were from Sigma Merck (St Louis, MO, USA). The purity of these reagents was at least 95%, procured by the manufacturer. Dihydrosanguinarine was obtained from BOC Sciences (Shirley, NY, USA) and had a purity of 98% according to the supplier. ACS-grade dichloromethane was obtained from Karal (León, Guanajuato, México).

### 4.2. Plant Material

Samples of *A. ochroleuca* were collected from the community of “El establo” (20°57′15.9″ N, 101°17′01.1″ W), Puentecillas, municipality of Guanajuato (Guanajuato, México) in March 2019. Dr. Eleazar Carranza from the herbarium Isidro Palacios (SLPM) identified and preserved the plant material (voucher number 53882).

### 4.3. Preparation of Plant Extract and Sample Treatment

Shade-dried and finely mechanically triturated stems of *A. ochroleuca* (2200 g) were extracted by maceration in dichloromethane (10 L) at 25 °C for 72 h, protected from light. Dichloromethane was eliminated from the extract by distillation at reduced pressure, obtaining 8.3 g of dry extract (yield = 37.7%).

### 4.4. Sample Treatment and GC–MS Analysis 

Fifty milligrams of the extract were dissolved in 2.5% hydrochloric acid (5 mL). This aliquot was filtered, and the pH of the solution was adjusted to 8 with a concentrated ammonium hydroxide solution and extracted with dichloromethane (3 × 5 mL). The extracts were dried and resuspended in 500 µL dichloromethane. The flow rate of helium, the carrier gas, was maintained at 1 mL/min, the injection volume was 1 µL, and the injection port was set to split 1:5 and maintained at 250 °C. The column oven temperature program was as follows: the initial temperature was held for 2 min, then the temperature was increased to 300 °C at 12 °C/min and held for 6 min (the total run time was 24.6 min). Temperature settings for the transfer line heater and ion source of the mass spectrometer were 290 °C and 250 °C, respectively. Spectra were acquired in the *m*/*z* range of 80–550; the filament was turned on after a solvent delay of 7 min, and the analysis was performed in Full Scan mode. A Bruker MS Workstation 8.0 was used for data collection, processing, and GC–MS control. The AMDIS software (Automated Mass Spectral Deconvolution and Identification System; http://www.amdis.net/, accessed on 5 April 2023) analyzed data in “Use Retention Index Data” mode. The identification of compounds was made through MS Search 2.0 software using NIST/EPA/NIH (NIST 2020) and Wiley 12th edition Mass Spectral Libraries.

### 4.5. Animals

Male Balb/c mice (weighing between 25 to 32 g, 6–8 weeks of age) from the bioterium of the Natural and Exact Sciences Division (University of Guanajuato) were housed in propylene cages in a room with a 12 h light/dark cycle, at 26 ± 2 °C and 50–60% relative humidity, with food (Laboratory Rodent Diet #5001, LabDiet, St. Louis, MO, USA) and water *ad libitum*. The Institutional Committee on Bioethics in Research of the University of Guanajuato (CIBIUG-P03-2020) approved all animal procedures.

### 4.6. Pharmacological Treatment

Trained observers performed the experiments in a silent room between 9 a.m. and 3 p.m. Different groups were assigned (n = 7 mice per group), including the vehicle (saline solution and polyvinylpyrrolidone (PVP; 4:1), 1.5 mg/kg clonazepam (CNZ) or 20 mg/kg fluoxetine (FLX) (positive controls), AOE (0.1, 1, 10, and 50 mg/kg p.o.), or DHS (0.1, 1, and 10 mg/kg p.o.). The plant extract or DHS was solubilized in saline solution and PVP (4:1), and 100 µL was administered to each mouse. Each test was conducted 1 h after post-treatment administration. Preliminary pharmacological work carried out in the laboratory and the results obtained from the acute toxicity test conducted in this study on the AOE, as well as previous toxicological studies performed with DHS [20,23] allowed for the selection of doses for the AOE and DHS. The ED_50_ value (the dose of AOE or DHS necessary to reach 50% of the pharmacological effect) was determined using linear regression analysis.

### 4.7. Acute Toxicity

The procedure was conducted according to the protocol described in [43]. Mice were treated with AOE (10–2000 mg/kg p.o.) and monitored for signs of toxicity (i.e., immobility, piloerection, anorexia, unusual respiratory pattern, etc.) and mortality every 24 h for 14 days.

### 4.8. Anxiolytic-like Tests

#### 4.8.1. Hole Board Test

A flat, closed platform (42 cm × 42 cm × 30 cm) with 16 spaced holes (3 cm in diameter) was used. The structure was 20 cm-high. At the beginning of each experiment, every rodent was in the central section of the board, and the number of dippings was counted for 5 min [28]. The anxiolytic-like mechanism of action of DHS was evaluated using 0.7 mg/kg bicuculline (a GABA_A_ receptor antagonist) or 1 mg/kg ketanserin (a 5-HT_2_ selective receptor antagonist). Each inhibitor was intraperitoneally administered 15 min before administering 10 mg/kg DHS. After 45 min, the hole board test was executed.

#### 4.8.2. Exploratory Cylinder Test

Each naïve mouse was placed into the Plexiglas cylinder, set in a vertical position. The experiment lasted 5 min and the number of rearings onto the posterior limbs of each mouse was registered [27]. The anxiolytic-like mechanism of action of the AOE was evaluated using similar neurotransmission inhibitors and similar conditions as described in Section 4.8.1

### 4.9. Antidepressant-like Tests

#### 4.9.1. Tail Suspension Test

Every mouse was suspended 40 cm above the ground for 6 min by fixing the tail with adhesive tape [29]. Immobility was measured when mice were passive and completely motionless. The last 4 min of the experiment were recorded.

#### 4.9.2. Forced Swimming Test

Plexiglas cylinders were filled to 40% of their capacity with water at 25 °C. The time (in sec) of immobility, considered when each mouse was floating with its nose above water, was registered for 6 min [30]. Every test lasted 6 min, but only the last 4 min of the test were recorded. In additional experiments, mice were pre-treated with 0.05 mg/kg prazosin (an α1-adrenoreceptor blocker), 1 mg/kg atropine (a muscarinic cholinergic antagonist), or 3 mg/kg cyproheptadine (H1 and 5-HT_2B_ receptor antagonist) 15 min before the administration of the AOE. After 45 min, the forced swimming test was carried out.

### 4.10. Sedative and Locomotion Tests

#### 4.10.1. Pentobarbital-Induced Sedation Test

Mice were intraperitoneally injected with 50 mg/kg pentobarbital, a hypnotic dose, and the onset of sleep (from the injection of pentobarbital until the loss of locomotor activity) and the duration of sleep (loss of locomotor activity until the recovery of righting reflex) were registered [31].

#### 4.10.2. Rotarod Test

Mice were trained daily for three days. Animals capable of continuing to walk on the rotarod (Harvard Apparatus, Barcelona, Spain), set at 4 revolutions per min for 4 min, were selected for the experiment. The permanence (in sec) on the rotarod was recorded at 60 and 120 after treatment [32]. The cut-off time was 4 min.

### 4.11. Anticonvulsant Effect

The induced-convulsion compounds strychnine (2 mg/kg i.p.) and 4-aminopyridine (10 mg/kg i.p.) were administered to mice after 60 min of drug treatment. Animals were placed in Plexiglas cylinders to record the number of deaths, the onset, and the duration of convulsions [33]. To evaluate a possible mechanism of action, animals were pre-treated with 2 mg/kg flumazenil (a GABA_A_ antagonist) 15 min before administering 1 mg/kg p.o. AOE. After 45 min, the strychnine-induced convulsion test was conducted. Mortality (%) was calculated as follows: (number of dead animals/total number of animals) × 100, whereas protection against seizures was estimated as follows: (number of live animals/total number of animals) × 100. Live animals were considered those mice that remained alive 24 h after the induction of convulsions.

### 4.12. Molecular Docking Studies

AutoDock Vina [44] was used for docking to predict the binding affinity of dihydrosanguinarine to the GABA_A_ receptor (PDB id: 6D6T, with a resolution of 3.86 Å) and α_2A_-adrenergic receptor (PDB id: 6KUX, with a resolution of 2.70 Å). The MOE 2022.022 software [45] protonated the GABA_A_ and α_2A_-adrenergic receptors and dihydrosanguinarine structure. Each grid was centered at the crystallographic coordinates of ethyl 8-fluoro-5-methyl-6-oxo-5,6-dihydro-4*H*-imidazo[1,5-a][1,4]benzodiazepine-3-carboxylate (FYP; center_x = 119.613, center_y = 169.091, and center_z = 154.264) and (8~{a}~{R},12~{a}~{S},13~{a}~{S})-12-ethylsulfonyl-3-methoxy 5,6,8,8~{a},9,10,11,12~{a},13,13~{a}-decahydroisoquinolino[2,1-g][1,6]naphthyridine (E3F; center_x = −2.729, center_y = −7.885, and center_z = −19.147) of GABA_A_ and α_2A_-adrenergic receptor, respectively. The grid dimensions were 25 × 20 × 20 points and 20 × 20 × 20 points with a default spacing. PyMOL v1.9 software (The PyMol Molecular Graphics System, version 1.9 Schrödinger, LCC.) and Discovery Studio Visualizer, version 21.1.0.20298, were used for obtaining all visualizations.

### 4.13. Calculation of ADMET Properties

The pharmacokinetic parameters of the compounds DHS, FYP, and E3F were calculated using https://tox-new.charite.de/protox_II/, accessed on 3 August 2023 and http://www.swissadme.ch/index.php, accessed on 2 August 2023. This information is required in order to optimize the pharmacodynamic response and the extent of availability as a function of the route of administration.

### 4.14. Statistical Analysis

The Shapiro–Wilk test was used to evaluate the normal distribution. The results were analyzed by one-way ANOVA followed by Dunnett’s test and considered statistically significant if *p* < 0.05. When necessary, a t-student test was carried out. Data analysis was performed using Statistica software, version 10 (Statsoft, Tulsa, OK, USA).

## 5. Conclusions

The AOE exerted anxiolytic, antidepressant, and anticonvulsant actions. The AOE induced anxiolytic-like and anticonvulsant effects due to GABAergic participation, and the antidepressant-like effects were mediated by the noradrenergic system. DHS induced anxiolytic-like activity due to GABAergic and noradrenergic participation.

## Figures and Tables

**Table 1:** List of compounds detected in AOE.

Classification	Metabolite Name	Retention Time
Aliphatic compounds	3-methyltridecane	8.0
	2,6,10-trimethyldodecane	8.3
	2-dodecanone	8.3
	3-methyltetradecane	9.5
	Hexadecane	9.9
	4-methylhexadecane	10.0
	3-methypentadecane	10.0
	2,6,10-trimethylpentadecane	10.4
	Octadecane	10.7
	2,6,11,15-tetramethylhexadecane	11.2
	2,6,10,14-tetramethylhexadecane	11.3
	2-methyloctadecane	11.9
	Nonadecane	12.6
	Eicosane	13.0
	Heneicosane	14.1
	Docosane	14.7
	Tricosane	15.8
Alkaloids	Canadine (Tetrahydroberberine)	20.6
	Stylopine (Tetrahydrocoptisine)	20.7
	Allocryptopine	20.8
	Protopine	20.9
	Cryptopalmatine (Muramine)	21.0
	(±)-Corynoline	21.0
	Dihydrosanguinarine	22.3
	Dihydrochelerythrine	22.5
Aromatic compounds	*p*-Diacetylbenzene	8.2
Carboxylic acids and related compounds	Dodecyl acrylate	10.9
	Tetradecanoic acid	11.3
	Hexadecanoic acid methyl ester	12.8
	14-methyl pentadecanoic acid methyl ester	12.8
	10,13-dimethyltetradecanoic acid methyl ester	12.8
	Hexadecanoic acid	13.1
	Octadecanoic acid	14.7
Phenolic compounds	Vanillin	7.8
	2,4-di-tert-butylphenol	8.9
	8-hydroxy-3-methyl-3,4-dihydro-1*H*-2-Benzopyran-1-one	9.4
	4,5-dimethoxy-hydroxybenzoic acid methyl ester	11.5
	Ferulic acid methyl ester	12.1
	Benzenepropanoic acid, 3,5-bis(1,1-dimethylethyl)-4-hydroxy-, methyl ester	12.9
Benzoquinones and related compounds	7,9-di-tert-butyl-1-oxaspiro[4.5]deca-6,9-diene-2,8-dione	12.7
Terpenes	Dihydroactinolide	9.3
	Isozizanoic acid	11.4
	Dihydrofrullanolide	11.4
	(-)-Loliolide	11.6

**Table 2:** Effect of AOE on mice treated with 2 mg/kg strychnine or 4-aminopyridine-induced convulsions. Data are presented as mean ± standard error of the mean (n = 7 per group), ** *p* < 0.05 vs. the vehicle group.

Treatment	Onset of Convulsion (s)	Duration of Convulsion (s)	Mortality (%)	Protection against Seizures (%)
**2 mg/kg strychnine**				
Vehicle	130.3 ± 14.6	40.2 ± 3.9	100	0
CNZ 1.5 mg/kg	201.2 ± 6.8 **	20.9 ± 3.1 **	80	20
AOE 0.1 mg/kg	185.4 ± 8.3	36.1 ± 3.8	60	40
AOE 1 mg/kg	221.3 ± 16.7 **	40.9 ± 3.7	40	60
AOE 10 mg/kg	240.8 ± 18.9 **	34.5 ± 2.3	0	100
1 mg/kg AOE + 2 mg/kg flumazenil	140.1 ± 3.2	42.3 ± 2.3	100	0
**10 mg/kg 4-aminopyridine**				
Vehicle	129 ± 4.2	42.1 ± 3.3	100	0
CNZ 1.5 mg/kg	490.1 ± 15.6 **	15.8 ± 1.3 **	70	30
AOE 0.1 mg/kg	265 ± 7.3	39.2 ± 2.9	100	0
AOE 1 mg/kg	334.5 ± 7.4 **	36.4 ± 1.7	100	0
AOE 10 mg/kg	461.8 ± 4.9 **	30 ± 0.9	100	0
DHS 0.1 mg/kg	114.8 ± 17.4	143.0 ± 23.7 **	100	0
DHS 1 mg/kg	125.2 ± 15.6	208.4 ± 43.4 **	100	0
DHS 10 mg/kg	184.6 ± 14.6 **	180.0 ± 09.1 **	100	0

** *p* < 0.05, compared to the vehicle group.

**Table 3:** ADMET properties of DHS.

Model	Property	Result		
Absorption		DHS	FYP	E3F
Blood–Brain Barrier	Permeability	Yes	Yes	Yes
Human Intestinal Absorption	High	Yes	Yes	Yes
P-glycoprotein	Substrate	Yes	No	Yes
**Metabolism**				
CYP450 1A2	Affinity	Yes	No	No
CYP450 2C9	Affinity	Yes	No	No
CYP450 2D6	Affinity	No	No	No
CYP450 2C19	Affinity	Yes	No	No
CYP450 3A4	Affinity	Yes	No	No
**Toxicity**				
Hepatotoxicity	Inactive	0.75	0.95	0.83
Cytotoxicity	Inactive	0.71	0.57	0.56
Lethal dose 50	mg/kg	500	1300	74
Toxicity category	Class	IV	IV	I

## Data Availability

Not applicable.
